# Silencing of Cholesterol 25‐Hydroxylase Attenuates Lipopolysaccharide‐Induced Cardiomyocyte Damage In Vitro

**DOI:** 10.1111/jcmm.70959

**Published:** 2025-11-26

**Authors:** Yi‐jiao Men, Hong‐bo Cheng, Yan‐ling Dong, Yu Gong, Ya‐qing An, Ying‐li Jin, Shu‐na Hao, Yu Ma, Ying‐ping Tian

**Affiliations:** ^1^ Department of Emergency The Second Hospital of Hebei Medical University Shijiazhuang China

**Keywords:** apoptosis, CH25H, mitochondrial dysfunction, oxidative stress, sepsis‐induced myocardial dysfunction

## Abstract

The underlying mechanisms of sepsis‐induced myocardial dysfunction (SIMD) remain elusive, and no targeted therapies currently exist. This study aimed to explore the expression features and functional effects of cholesterol 25‐hydroxylase (CH25H) in SIMD in vitro. CH25H was identified as an upregulated gene related to SIMD through bioinformatics analysis. Its upregulation was validated in the myocardial tissue of SIMD mice as well as in lipopolysaccharide (LPS)‐induced primary cardiomyocytes and AC16 cells. CH25H overexpression elevated 25‐hydroxycholesterol levels and aggravated oxidative stress, mitochondrial dysfunction, apoptosis, and NOD‐like receptor family pyrin domain containing 3 (NLRP3) inflammasome and NF‐κB pathway activation in AC16 cells. The effect of CH25H overexpression was similar to that induced by LPS treatment. Conversely, silencing CH25H attenuated these LPS‐induced injuries. Furthermore, CH25H overexpression exacerbated oxidative stress, mitochondrial dysfunction, and apoptosis in LPS‐stimulated AC16 cells, and these effects of CH25H overexpression can be counteracted by the NLRP3 inhibitor. In conclusion, CH25H may promote LPS‐induced cardiomyocyte injury through NLRP3/NF‐κB pathway activation.

**Abbreviations**
25‐HC: 25‐hydroxycholesterol
ASC: apoptosis‐associated speck‐like protein containing a CARD
ATP: adenosine triphosphate
BAX: BCL2‐associated X
BCL: B‐cell lymphoma
CAT: catalase
CH25H: cholesterol 25‐hydroxylase
CH25H‐OE: CH25H overexpression plasmid
CLP: cecal ligation and puncture
cTnT: cardiac troponin T
DAB: 3,3′‐diaminobenzidine
DEG: differentially expressed genes
ELISA: enzyme‐linked immunosorbent assay
FCGR2B: Fc gamma receptor IIb
GEO: Gene Expression Omnibus
HE: haematoxylin and eosin
IHC: immunohistochemistry
IL‐6: interleukin‐6
LPS: lipopolysaccharide
LVEF: left ventricular ejection fraction
LVFS: left ventricular fractional shortening
MDA: malondialdehyde
MT‐ND1: mitochondrially encoded NADH dehydrogenase 1
NLRP3: NOD‐like receptor family pyrin domain containing 3
NT‐proBNP: N‐terminal pro B‐type natriuretic peptide
OGD/R: oxygen–glucose deprivation and reoxygenation
PBS: phosphate‐buffered saline
PCM: primary cardiomyocytes
qRT‐PCR: fluorescence quantitative reverse transcription PCR
ROS: reactive oxygen species
si‐CH25H: SiRNA targeting CH25H
SIMD: sepsis‐induced myocardial dysfunction
si‐NC: negative control siRNA
siRNA: small‐interfering RNA
T2DM: type 2 diabetes mellitus
TEM: transmission electron microscopy
TNF‐α: tumour necrosis factor alpha
TUNEL: terminal deoxynucleotidyl transferase dUTP nick end labeling

## 1 Introduction

Sepsis is a systemic inflammatory response syndrome caused by pathogenic microorganisms [1]. Sepsis‐induced myocardial dysfunction (SIMD) is a severe complication of sepsis [2]. Clinically, it is characterised by heart dysfunction, including ventricular dilation, impaired contractility, and reduced ejection fraction [2, 3]. The mechanisms underlying SIMD include excessive inflammation, metabolic disturbances, cardiomyocyte apoptosis, oxidative‐nitrosative stress, mitochondrial dysfunction, and autonomic dysregulation [4]. SIMD significantly affects the outcome of septic shock [3]. However, its mechanism of action remains unclear, and no specific treatment is available. Further investigation of the cellular and molecular mechanisms underlying SIMD is crucial for accurate diagnosis, targeted therapy, and improved patient outcomes.

Cholesterol 25‐hydroxylase (CH25H) plays a crucial role in lipid metabolism [5]. It catalyses the oxidation of cholesterol to produce the soluble product 25‐hydroxycholesterol (25‐HC). In addition to lipid metabolism, CH25H is involved in regulating inflammation, immune responses, and antiviral defence [5, 6]. CH25H is also important in regulating pancreatic cancer, melanoma, lung cancer, and colon cancer [7, 8, 9, 10, 11]. CH25H plays distinct functional roles in cardiac pathology [12, 13]. Its expression significantly increases in H9C2 cardiomyocytes under oxygen–glucose deprivation and reoxygenation (OGD/R) conditions [12]. Knockdown of CH25H inhibits OGD/R‐induced NOD‐like receptor protein 3 (NLRP3) inflammasome activation and pyroptosis [12]. Furthermore, CH25H and 25‐HC levels are notably lower in mice with type 2 diabetes mellitus (T2DM), and CH25H knockout exacerbates cardiac dysfunction in T2DM models [13]. However, the precise expression pattern of CH25H and its underlying molecular mechanisms in SIMD remain unclear and require further investigation.

In this study, we conducted a bioinformatics analysis of SIMD‐related datasets and identified CH25H as a potential key gene in septic myocardial tissue. We then validated CH25H expression in the myocardial tissue of SIMD mice, primary cardiomyocytes (PCM), and AC16 cells exposed to lipopolysaccharide (LPS). We also explored the effects of CH25H on LPS‐induced oxidative stress, mitochondrial function, apoptosis, and NLRP3 inflammasome activation at the cellular level. We aimed to provide a new experimental foundation in vitro for identifying potential targets for the clinical treatment and evaluation of SIMD.

## 2 Materials and Methods

### 2.1 Bioinformatic Prediction

The keywords “sepsis” and “heart” were used to search the Gene Expression Omnibus (GEO) database, and three datasets (GSE153086, GSE40180, and GSE53007) were selected for analysis. Differentially expressed genes (DEGs) in septic myocardial tissues were identified using GEO2R with thresholds of |logFC| > 1.5 and adjusted *p*‐value < 0.05. Common DEGs across all three datasets were identified using Venn diagram analysis. Finally, these common DEGs were subjected to functional enrichment analysis, including Gene Ontology (GO) terms and Kyoto Encyclopedia of Genes and Genomes (KEGG) pathways.

### 2.2 Construction of a Mouse Sepsis Model

Eighteen C57BL/6 mice were randomly divided into three groups: control, sham surgery, and sepsis (*n* = 6). A sepsis model was established using cecal ligation and puncture (CLP) [14]. Mice were monitored every 6 h for 24 h. Cardiac systolic function and heart rate were evaluated using a high‐resolution ultrasound system (Vevo 2100 Imaging System, FUJIFILM VisualSonics Inc., Toronto, Canada) equipped with an MS400 transducer (frequency range: 18–38 MHz). All examinations were performed by an experienced operator who was blinded to the experimental groups. Mice with a significant decrease in left ventricular ejection fraction (LVEF) and left ventricular fractional shortening (LVFS) were classified as the SIMD group (*n* = 6). In the sham surgery group (*n* = 6), a laparotomy was performed without ligation or puncture. Untreated mice were classified as the control group (*n* = 6). At 24 h after CLP, the animals were anaesthetised, blood was collected from the eyeball, and serum was separated for an enzyme‐linked immunosorbent assay (ELISA). Mice were then sacrificed by cervical dislocation. The hearts of three mice from each group were perfused with 4% paraformaldehyde, and their left ventricles were collected and fixed in 4% paraformaldehyde for haematoxylin and eosin (HE) staining, terminal deoxynucleotidyl transferase dUTP nick end labeling (TUNEL) assay, and immunohistochemistry (IHC) analysis. The left ventricles of other mice were collected and frozen in liquid nitrogen for fluorescence quantitative reverse transcription PCR (qRT‐PCR).

### 2.3 HE Staining and Masson's Trichrome Staining

Fixed myocardial tissues (*n* = 3) were embedded and sectioned. Sections were deparaffinised and dehydrated. For HE staining, the sections were stained with haematoxylin for 10 min, rinsed, treated with 1% hydrochloric acid alcohol, and blued with running water. The sections were then stained with eosin for 1 min, rinsed, and dehydrated in 95% ethanol and anhydrous ethanol. Finally, the sections were cleared in xylene and mounted on coverslips for examination. Masson staining was performed using Masson's trichrome stain kit (G1340, Solarbio, Beijing, China). Histopathological assessment was conducted by two experienced pathologists who were blinded to the experimental groups. Pathological scores were determined by consensus.

### 2.4 TUNEL

Apoptotic cells in paraffin‐embedded myocardial tissues (*n* = 3) were measured using a TUNEL In Situ Apoptosis Kit (Horseradish peroxidase‐3,3′‐Diaminobenzidine [HRP‐DAB] Method; E‐CK‐A331, Elabscience, Wuhan, China) according to the manufacturer's instructions.

### 2.5 ELISA and Mitochondrial Complex I Activity Assay

ELISA kits for mouse interleukin‐6 (IL‐6, MB‐2899A), tumour necrosis factor alpha (TNF‐α, MB‐2868A), N‐terminal pro B‐type natriuretic peptide (NT‐proBNP, MB‐3294A), and cardiac troponin T (cTnT, MB‐5879A) were purchased from Jiangsu Meibiao Biotechnology Co. Ltd. (Yancheng, China). Cell mitochondrial complex I activity assay kit was purchased from Elabscience Biotechnology Co. Ltd. (E‐BC‐K834‐M). Human 25‐HC ELISA kit was purchased from MyBioSource (MBS7254215, San Diego, CA, USA). These assays were performed in three independent experiments, each with three technical replicates.

### 2.6 qRT‐PCR

Total RNA was extracted using TRIzol reagent (R401‐01, Vazyme, Nanjing, China). cDNA was synthesised by reverse transcription using a HiScript II 1st Strand cDNA Synthesis Kit (R211‐02, Vazyme). The PCR mixture was prepared according to the protocol of the AceQ qPCR SYBR Green Master Mix kit (Q121‐02, Vazyme), and PCR was performed using a LongGene Q2000B Fluorescence Quantitative PCR Instrument (Hangzhou, China). CH25H and Fc gamma receptor IIb (FCGR2B) expression levels were calculated using the 2^−△△CT^ method. The primer sequences were CATCCTGCGACGCTACAAGA and GCGAAGATCTCGGTGTCGAA for CH25H, AGGAGAACAATTAATGTTAGGATGC and AGAAGCATCATCTTTGACAGGT for FCGR2B, and GTCCCTCACCCTCCCAAAAG and GCTGCCTCAACACCTCAACCC for the reference gene beta‐actin. qRT‐PCR was performed in three independent experiments.

### 2.7 IHC

Myocardial tissue sections from three groups (control, sham surgery, and SIMD; *n* = 3 animals per group) were first deparaffinised and rehydrated, and then dried overnight in a 60°C oven. Antigen retrieval was performed by heating sections in 0.01M sodium citrate buffer in a pressure cooker for 3 min, followed by cooling and washing. Endogenous peroxidase activity was blocked with 3% H_2_O_2_ at room temperature for 10 min, and the sections were washed with phosphate‐buffered saline (PBS). Sections were blocked with 5% bovine serum albumin for 15 min and incubated with a diluted anti‐CH25H antibody (1:300; ab214295, Abcam, Cambridge, MA, USA) or anti‐CD31 antibody (1:10,000; 28083‐1‐AP, Proteintech, Wuhan, China) overnight at 4°C. After washing with PBS, the sections were incubated with a diluted horseradish peroxidase‐conjugated secondary antibody (Gene Tech, Wuhan, China) for 1 h. DAB staining was performed for 5 min and terminated by washing with water. Haematoxylin counterstaining, differentiation with 1% hydrochloric acid in ethanol, and blueing in running water were subsequently performed. The sections were dehydrated using graded ethanol, immersed in xylene, and mounted with neutral gum for microscopy and imaging. The expression level of CH25H was quantified by two experienced pathologists, who were blinded to the experimental groups, using the same evaluation standard reported by other researchers [15].

### 2.8 Mouse PCM Isolation

Mouse PCM was isolated from postnatal heart according to the method reported by Feng et al. [16]. AC16 cells were purchased from Shanghai Anwei Biotechnology Co. Ltd. (Shanghai, China) and cultured according to the manufacturer's instructions.

### 2.9 Cell Treatment

The full‐length coding sequence of mouse CH25H was cloned into the pcDNA3.1(+) plasmid to construct the CH25H overexpression plasmid (CH25H‐OE). A small interfering RNA (siRNA) targeting CH25H (si‐CH25H) was purchased from Guangzhou Dongze Biotechnology Co. Ltd. The sequences of the three si‐CH25H and negative control siRNAs (si‐NC) were as follows: si‐CH25H‐1, ccuucgugguccuggauautt; si‐CH25H‐2, guaugagcgucugggaatt; si‐CH25H‐3, gcuacaagauccacccugatt; and si‐NC, uucuccgaacgugugucacgutt. To determine the optimal LPS concentration, AC16 cells were treated with different concentrations of LPS (0, 5, 10, 20, and 30 μg/mL). After 24 h of treatment, cell viability was assessed using the CCK‐8 assay (CK04, Dojindo, Japan), and the concentrations of TNF‐α and IL‐6 in the supernatant were measured by ELISA. To measure the effect of LPS treatment on CH25H expression, PCM and AC16 cells were treated with or without 20 μg/mL LPS for 24 h. To investigate the role and mechanism of CH25H in regulating LPS‐induced injury, AC16 cells were divided into the following groups: control group, untreated; LPS group, treated with 20 μg/mL LPS; empty vector group, transfected with pcDNA3.1(+) plasmid; CH25H‐OE group, transfected with CH25H‐OE; LPS + si‐NC group, transfected with si‐NC and treated with 20 μg/mL LPS; LPS + si‐CH25H group, transfected with si‐CH25H and treated with 20 μg/mL LPS; LPS + empty vector group, transfected with pcDNA3.1(+) plasmid and treated with 20 μg/mL LPS; LPS + CH25H‐OE group, transfected with CH25H‐OE and treated with 20 μg/mL LPS; LPS + MCC950 group, treated with 20 μg/mL LPS and 1 μM NLRP3 inhibitor MCC950; LPS + CH25H‐OE + vehicle group, transfected with CH25H‐OE and treated with 20 μg/mL LPS and DMSO; LPS + CH25H‐OE + MCC950 group, transfected with CH25H‐OE and treated with 20 μg/mL LPS and 1 μM MCC950. After 24 h of pre‐transfection, the cells were treated with LPS or MCC950 for 24 h, followed by the relevant assays.

### 2.10 Western Blot

Total protein was extracted from the cells, and its concentration was measured. The protein solution was mixed with loading buffer at a 2:1 ratio and heated for 5 min. Then, 30 μg of protein was loaded per well for sodium dodecyl sulfate–polyacrylamide gel electrophoresis. After electrophoresis, the proteins were transferred to a polyvinylidene fluoride membrane. After blocking, the membrane was incubated overnight at 4°C with the following primary antibodies: CH25H (1:1000; MBS9231078, MyBioSource), B‐cell lymphoma 2 (BCL‐2; 1:1000; ab196495, Abcam), B‐cell lymphoma‐associated X protein (BAX; 1:3000; ab32503, Abcam), mitochondrially encoded NADH dehydrogenase 1 (MT‐ND1; 1:1000; ab181848, Abcam), NLR family pyrin domain containing 3 (NLRP3; 1:1000; 15101T, Cell Signalling Technology, Danvers, MA, USA), apoptosis‐associated speck‐like protein containing a CARD (ASC; 1:1000; 13833T, Cell Signalling Technology), pro‐caspase‐1 (1:1000; #3866, Cell Signalling Technology), cleaved caspase‐1 (1:1000; 4199T, Cell Signalling Technology), IL‐1β (1:1000; 12242T, Cell Signalling Technology), inhibitor of nuclear factor kappa B alpha (IκBα; 1:1000; 4812T, Cell Signalling Technology), phosphorylated IκBα (p‐IκBα; 1:1000; 2859T, Cell Signalling Technology), nuclear factor kappa B p65 subunit (p65; 1:1000; BF8005, Affinity Biosciences, Nanjing, China), phosphorylated p65 (p‐p65; 1:1000; AF2006, Affinity Biosciences), and GAPDH (1:10,000; AF7021, Affinity Biosciences, Nanjing, China). Following washing with Tris‐buffered saline with Tween 20, the membrane was incubated for 1 h at 37°C with HRP‐conjugated goat anti‐rabbit immunoglobulin G (IgG) (H + L) (1:10,000; BA1054, Boster, Wuhan, China) or HRP‐conjugated goat anti‐mouse IgG (H + L) (1:10,000; BA1050, Boster). Finally, the membrane was rinsed with distilled water for 2 min, repeated three times. A chemiluminescent substrate was added to the membrane in a dark room, and signals were detected by X‐ray film exposure. Western blot was repeated three times independently.

### 2.11 Measurement of Oxidative Stress Indicators

Catalase (CAT) activity was measured using a CAT activity assay kit (E‐BC‐K031‐M, Elabscience). Malondialdehyde (MDA) content was determined using an MDA ELISA kit (E‐EL‐0060, Elabscience). To measure intracellular reactive oxygen species (ROS) concentrations, an ROS detection kit (S0033; Beyotime, Shanghai, China) was used to treat the cell samples, and the signals were detected using a flow cytometer (FACSCanto, BD Biosciences, Bedford, MA, USA). These assays were performed in three independent experiments.

### 2.12 Flow Cytometry Mitochondrial Membrane Potential (Δψ) Detection

Cells were stained with fluorescent probe JC‐1 according to the method of BD MitoScreen Kit (551302, BD Biosciences) and analysed by flow cytometry (FACSCanto, BD Biosciences). The ratio of red (FL2) to green (FL1) fluorescence intensity was calculated to determine Δψ status. Three independent biological replicates were performed.

### 2.13 Transmission Electron Microscopy (TEM) Sample Preparation for Mitochondrial Ultrastructure Analysis

Cell pellets from each group were collected and fixed with ice‐cold 2.5% glutaraldehyde solution at 4°C for 2 h. After fixation, the cells were washed with PBS to remove any residual glutaraldehyde. Subsequently, the samples were post‐fixed with 1% osmium tetroxide at 4°C for 1 h. Following post‐fixation, the cells were dehydrated using a graded series of ethanol or acetone solutions. The dehydrated samples were then embedded in resin and polymerised. Ultrathin sections were prepared and air‐dried. The sections were stained with uranyl acetate and lead citrate solutions for contrast enhancement. Finally, the samples were examined under a HT7800 TEM (Hitachi, Tokyo, Japan) for mitochondrial ultrastructure analysis. Three independent biological replicates were performed.

### 2.14 Measurement of Adenosine Triphosphate (ATP) Level

The cells were treated using an ATP assay kit (A095‐1‐1; Nanjing Jiancheng Bioengineering Institute, Nanjing, China). The absorbance of each tube was measured at 636 nm. ATP levels (μmol/gprot) were calculated using the method described in the kit based on absorbance values. This assay was performed in three independent experiments, each with three technical replicates.

### 2.15 Hoechst 33258 Staining

The culture medium from each group was removed, and the samples were washed 1–2 times with PBS. An appropriate amount of Hoechst 33258 staining reagent (BS116‐25 mg; Biosharp, Hefei, China) was added to fully cover the samples. The samples were then incubated in the dark at 37°C for 20–30 min. After incubation, the samples were washed twice with PBS. Finally, an adequate amount of PBS was added to cover the samples, and fluorescence was observed directly under a fluorescence microscope. This assay was repeated three times independently.

### 2.16 Statistical Analysis

Statistical analysis was performed using SPSS 19.0 software. The normality of data was confirmed by the Shapiro–Wilk test. Comparisons between two groups were performed using an independent t‐test, whereas comparisons among more than two groups were performed using one‐way ANOVA followed by Tukey's multiple comparisons test. Data are presented as mean ± standard deviation. Statistical significance was set at *p* < 0.05.

## 3 Results

### 3.1 DEGs in Septic Myocardial Tissue

**FIGURE 1:**
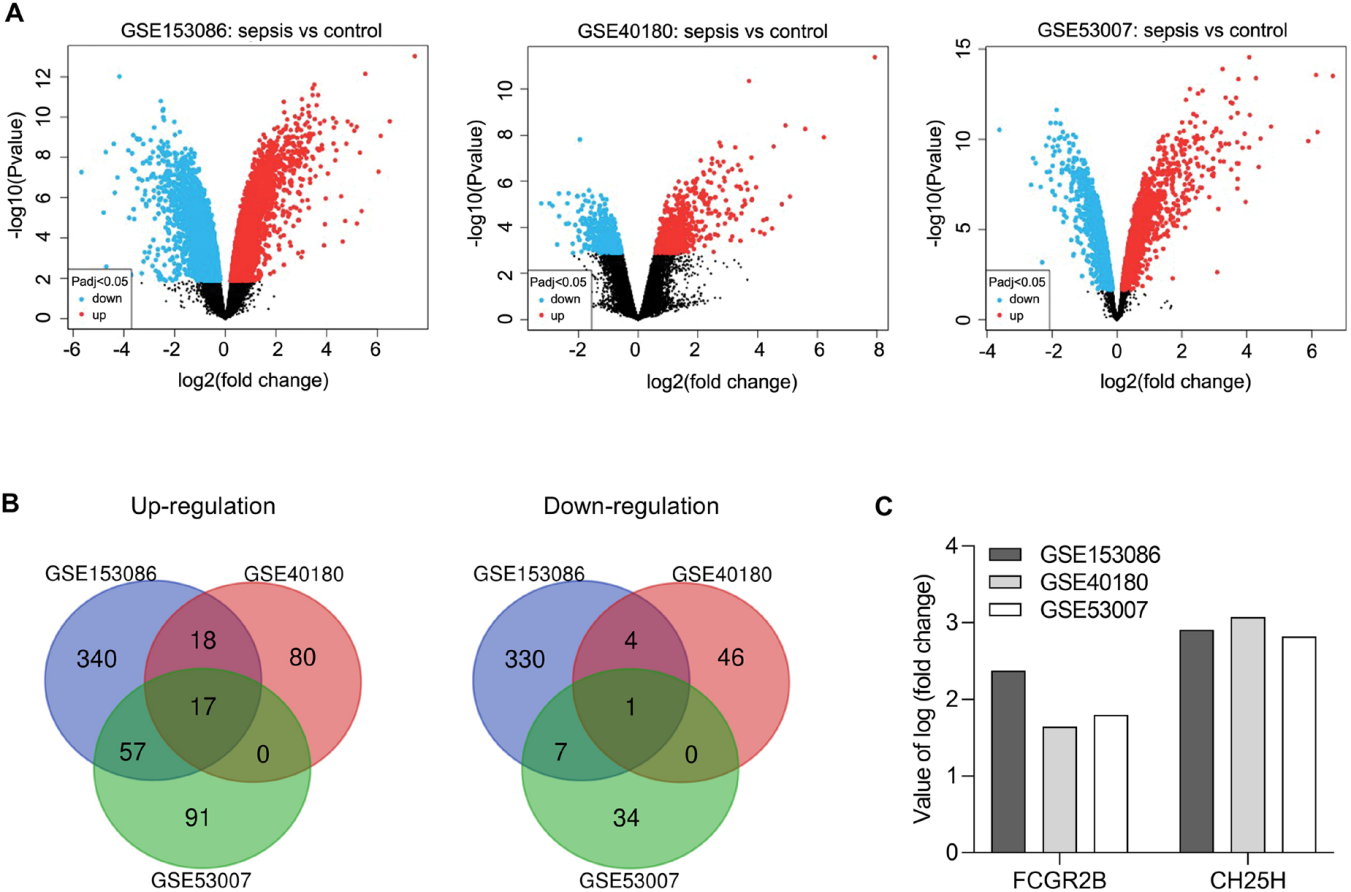
Identification of common differentially expressed genes in myocardial tissues from sepsis animal models using the data of three datasets (GSE153086, GSE40180, and GSE53007). (A) Volcano plots of three datasets. (B) Venn diagrams of upregulated and downregulated genes in the three datasets. (C) Value of log (fold change) of Fc gamma receptor IIb (FCGR2B) and cholesterol 25‐hydroxylase (CH25H) in the three datasets.

DEGs in septic myocardial tissue were identified using the data of three datasets (GSE153086, GSE40180, and GSE53007) (Figure 1A). One downregulated gene and 17 upregulated genes were identified using Venn analysis. Functional enrichment analysis of these DEGs, including GO and KEGG analysis, is presented in Figure S1. Genes with known functions in myocardial injury were excluded based on functional enrichment analysis and literature review. Therefore, FCGR2B and CH25H, being upregulated in all three datasets (Figure 1C), were selected for functional expression verification.

### 3.2 CH25H Is Highly Expressed in Myocardial Tissue of SIMD Mice

**FIGURE 2:**
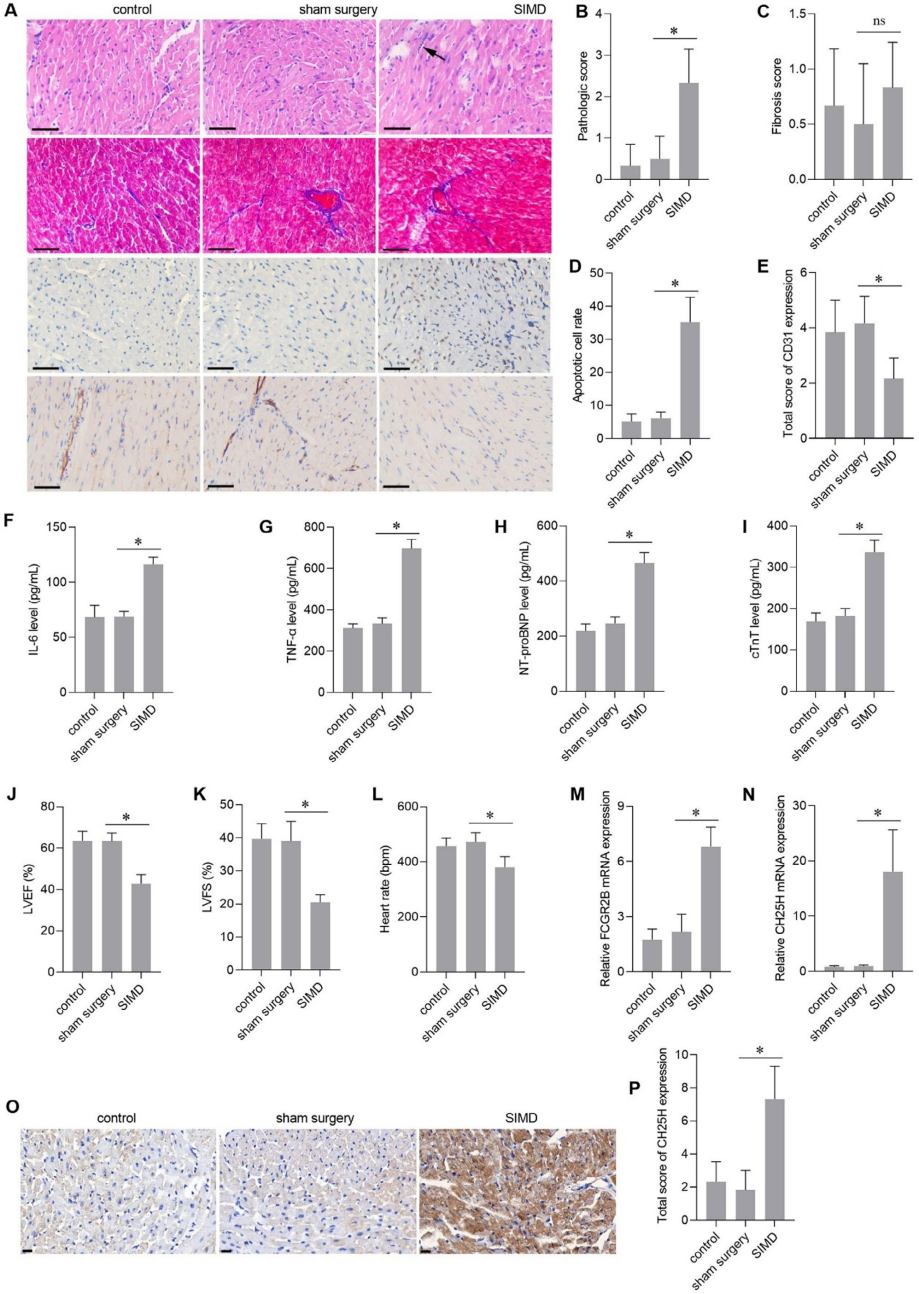
Fc gamma receptor IIb (FCGR2B) and cholesterol 25‐hydroxylase (CH25H) expression levels in myocardial tissue of sepsis‐induced myocardial dysfunction mice. (A) Representative images of haematoxylin and eosin (HE) staining, Masson's trichrome staining, terminal deoxynucleotidyl transferase dUTP nick end labeling (TUNEL), and immunohistochemistry (IHC) for CD31. The black arrow indicates inflammatory infiltration. Scale bar = 50 μm. (B) Pathological score based on HE staining. (C) Fibrosis area quantification based on Masson's trichrome staining. (D) Quantification of apoptotic cells by TUNEL assay. (E) Quantitative analysis of CD31 expression by IHC. (F–I) Interleukin‐6 (IL‐6), tumour necrosis factor alpha (TNF‐α), N‐terminal pro B‐type natriuretic peptide (NT‐proBNP), and cardiac troponin T (cTnT) levels in serum. (G–L) Value of left ventricular ejection fraction (LVEF), left ventricular fractional shortening (LVFS), and heart rate. (M and N) FCGR2B and CH25H mRNA expression levels. (O) Representative images of IHC for CH25H. (P) Statistical result of the total score of CH25H expression. Scale bar = 20 μm. *p < 0.05 and ^ns^ *p* > 0.05, *n* = 3.

The myocardial tissues of the SIMD group showed obvious morphological abnormalities, including disorganised arrangement, blurred cellular boundaries, and apparent nuclear staining heterogeneity with focal pallor (Figure 2A,B). Inflammatory infiltration was observed in the myocardial tissues of the SIMD group (black arrow, Figure 2A). TUNEL results showed an increased number of apoptotic cells in the myocardial tissues of the SIMD group (Figure 2A,D). The observed decrease in CD31 expression suggests a reduction in myocardial capillary density in the SIMD model (Figure 2A,E). In addition, the serum levels of IL‐6 (Figure 2F), TNF‐α (Figure 2G), NT‐proBNP (Figure 2H), and cTnT (Figure 2I) were significantly increased in the SIMD group. Moreover, significant decreases in LVEF (Figure 2J), LVFS (Figure 2K), and heart rate (Figure 2L) confirmed myocardial dysfunction in mice in the SIMD group. These results indicated that the SIMD model was successfully established. The mRNA levels of FCGR2B (Figure 2M) and CH25H (Figure 2N) in the myocardial tissues of the control, sham surgery, and SIMD groups were measured. The results showed that FCGR2B and CH25H mRNAs were markedly overexpressed in the myocardial tissues of SIMD mice, and the upregulation of CH25H was much greater than that of FCGR2B. FCGR2B is involved in the phagocytosis of immune complexes and in the regulation of antibody production by B cells. CH25H converts cholesterol to 25‐HC, which modulates multiple SIMD‐related pathological processes, including immune response, oxidative stress, mitochondrial dysfunction, and apoptosis [17, 18]. We hypothesised that CH25H may contribute to SIMD progression by modulating 25‐HC levels. Therefore, we chose CH25H for subsequent studies. Consistently, CH25H protein levels were obviously elevated in the myocardial tissues of the SIMD group (Figure 2O,P).

### 3.3 CH25H Is Upregulated in LPS‐Induced Cardiomyocyte

**FIGURE 3:**
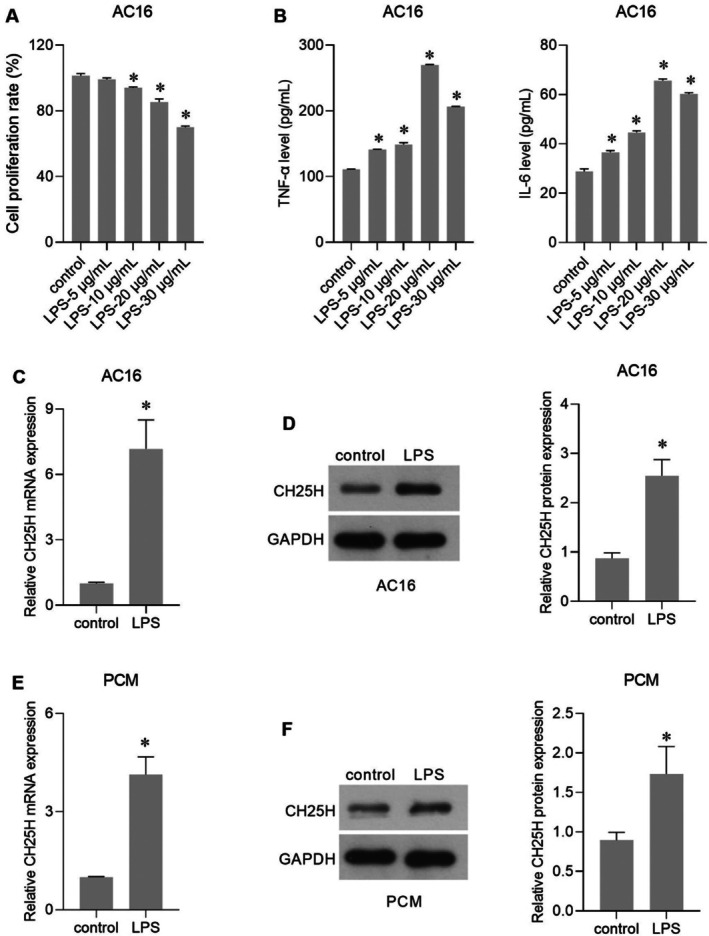
Cholesterol 25‐hydroxylase (CH25H) expression levels in lipopolysaccharide (LPS)‐induced AC16 cells and primary cardiomyocytes (PCM). (A) AC16 cell proliferation rate after treatment with different concentrations of LPS. (B) Tumour necrosis factor alpha (TNF‐α) and interleukin‐6 (IL‐6) levels in the supernatant of AC16 cells after treatment with different concentrations of LPS. (C, D) CH25H mRNA and protein expression levels in AC16 cells. (E, F) CH25H mRNA and protein expression levels in PCM. The right bar graphs in panels D and F show the statistical result of relative CH25H protein expression levels relative to GAPDH. *p < 0.05, *n* = 3.

As shown in Figure 3A,B, LPS treatment at 20 μg/mL significantly suppressed AC16 cell proliferation and had the most pronounced stimulatory effect on TNF‐α and IL‐6 production. Therefore, 20 μg/mL was chosen as the treatment concentration for the following assays. To verify the expression characteristics of CH25H at the cellular level, we constructed LPS‐induced PCM and AC16 cell models and measured their expression levels. LPS induction significantly increased CH25H mRNA and protein expression in both AC16 cells and PCM (Figure 3C–F), with more pronounced upregulation observed in AC16 cells. The effects of CH25H knockdown and overexpression on oxidative stress, mitochondrial function, and apoptosis were measured in AC16 cells.

### 3.4 Effect of CH25H on 25‐HC Level

**FIGURE 4:**
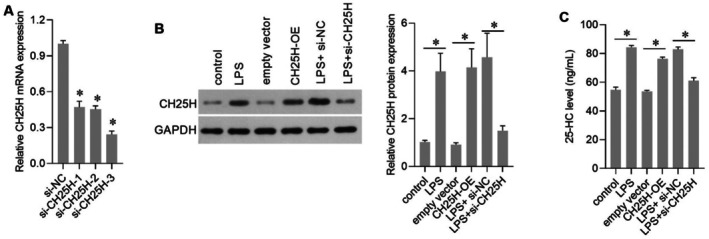
Cholesterol 25‐hydroxylase (CH25H) overexpression increased 25‐hydroxycholesterol (25‐HC) levels, and CH25H knockdown attenuated the effects of lipopolysaccharide (LPS) on 25‐HC. (A) CH25H mRNA levels in AC16 cells after transfection with three small interfering RNAs (siRNA) targeting CH25H (si‐CH25H‐1, si‐CH25H‐2, and si‐CH25H‐3). (B) CH25H protein levels. The bar graph on the right shows the expression level of CH25H relative to GAPDH. (C) 25‐HC levels. *p < 0.05, *n* = 3.

Three siRNAs were transfected into AC16 cells to silence CH25H expression. As shown in Figure 4A, si‐CH25H‐3 showed the highest silencing efficiency. Subsequently, si‐CH25H‐3 was termed si‐CH25H and chosen for other assays. The CH25H‐OE group was transfected with AC16 cells to overexpress CH25H (Figure 4B). In LPS‐treated AC16 cells, CH25H expression was successfully knocked down using si‐CH25H transfection (Figure 4B). Compared with the empty vector group, 25‐HC levels increased in the CH25H‐OE group (Figure 4C), indicating that CH25H overexpression enhanced 25‐HC production. This effect was similar to that induced by LPS treatment. Compared with the LPS + si‐NC group, 25‐HC levels decreased in the LPS + si‐CH25H group, indicating that silencing CH25H attenuated LPS‐induced excessive 25‐HC production in AC16 cells.

### 3.5 Effect of CH25H on Oxidative Stress in AC16 Cells

**FIGURE 5:**
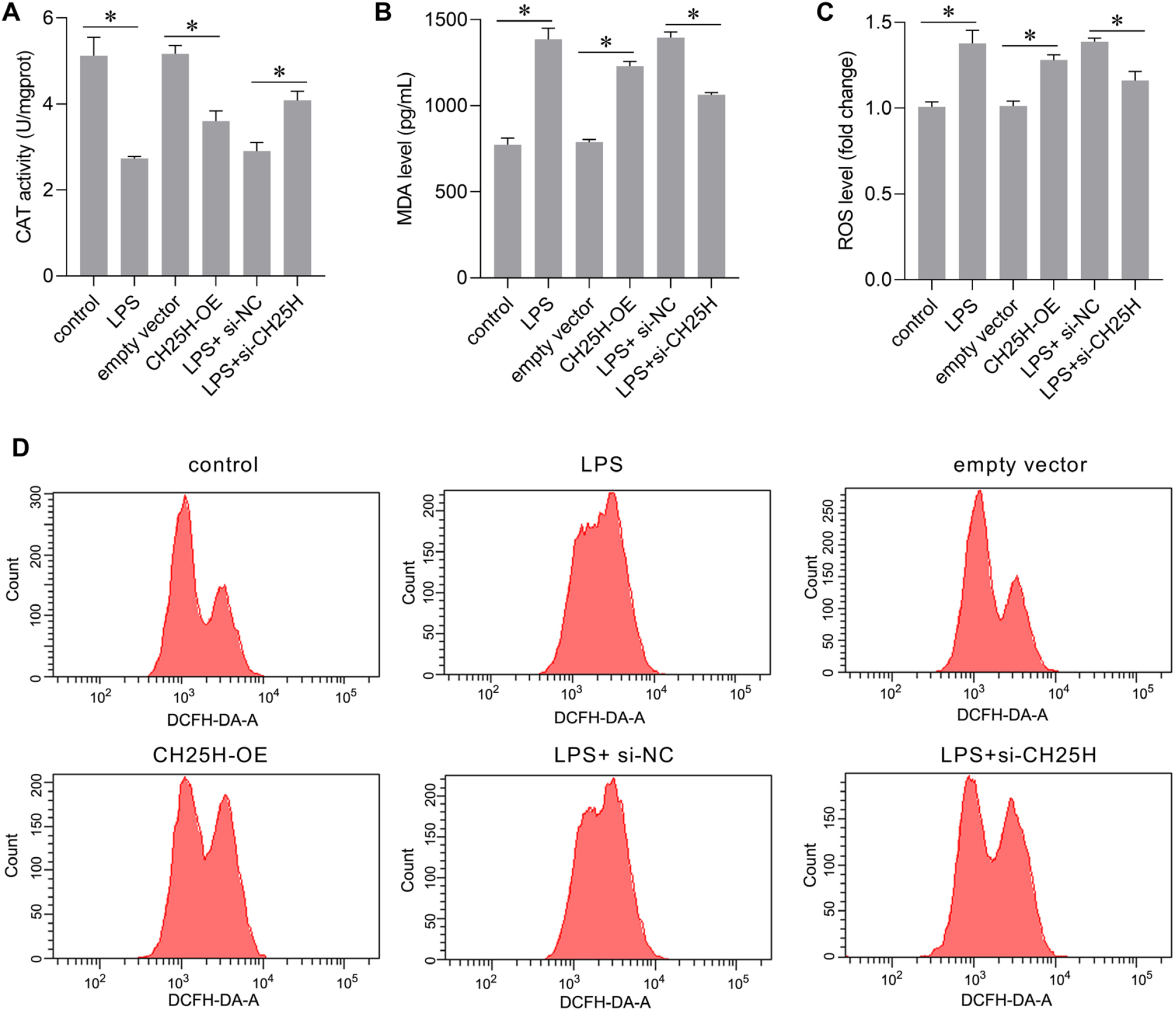
Cholesterol 25‐hydroxylase (CH25H) overexpression exacerbated oxidative stress, and CH25H knockdown attenuated the effects of lipopolysaccharide (LPS) on oxidative stress. (A–D) Effect of CH25H on oxidative stress markers (catalase [CAT], malondialdehyde [MDA], and reactive oxygen species [ROS]). Panel D shows representative flow cytometry images for ROS analysis. *p < 0.05, *n* = 3.

The effects of CH25H on oxidative stress were assessed by measuring CAT activity, and levels of MDA and ROS. Similar to the effects of LPS treatment, CH25H overexpression decreased CAT activity (Figure 5A) and increased MDA (Figure 5B) and ROS levels (Figure 5C,D). Compared with the LPS + si‐NC group, CAT activity increased (Figure 5A), whereas MDA (Figure 5B) and ROS levels (Figure 5C,D) decreased in the LPS + si‐CH25H group. These results show that CH25H overexpression aggravated oxidative stress, whereas silencing CH25H mitigated LPS‐induced oxidative stress in AC16 cells. However, CH25H knockdown did not affect oxidative stress in AC16 cells in the absence of LPS treatment (Figure S2A,B).

### 3.6 Effect of CH25H on Mitochondrial Function in AC16 Cells

**FIGURE 6:**
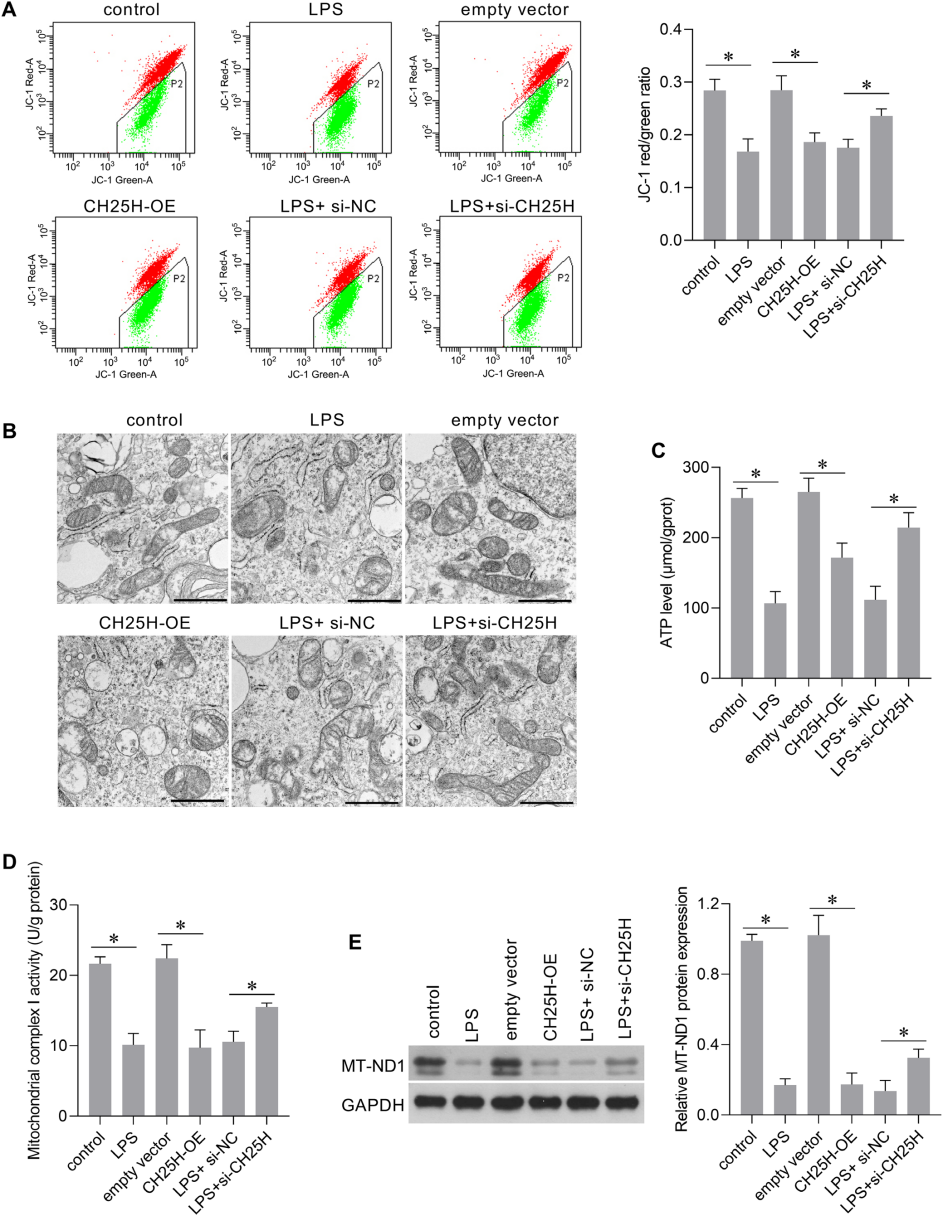
Cholesterol 25‐hydroxylase (CH25H) overexpression induced mitochondrial dysfunction, and CH25H knockdown attenuated the effects of lipopolysaccharide (LPS) on mitochondrial function. (A) The change of JC‐1 red/green ratio. (B) Transmission electron microscopy micrographs (scale bar = 1 μm). (C) ATP level. (D) Mitochondrial complex I activity. (E) Mitochondrially encoded NADH dehydrogenase 1 (MT‐ND1) protein level. The bar graph on the right shows the expression level of MT‐ND1 relative to GAPDH. *p < 0.05, *n* = 3.

Mitochondrial function is typically assessed using indicators such as ATP production, Δψ, and expression level or activity of mitochondrial‐related enzymes. Compared with the empty vector group, the JC‐1 red/green ratio decreased in the CH25H‐OE group, indicating that CH25H overexpression decreased the Δψ (Figure 6A). TEM micrographs revealed that empty vector cells had normal oval‐ or rod‐shaped mitochondria with intact double membranes and organised cristae, whereas CH25H‐OE cells showed swollen mitochondria with disrupted cristae, outer membrane discontinuity, and matrix leakage (Figure 6B). In addition, CH25H overexpression decreased ATP levels (Figure 6C), mitochondrial complex I activity (Figure 6D), and MT‐ND1 expression levels (Figure 6E), an essential component of complex I in the mitochondrial respiratory chain. However, CH25H knockdown did not affect ATP levels (Figure S2C), and mitochondrial complex I activity (Figure S2D) in AC16 cells without LPS treatment. These results indicated that CH25H overexpression induced mitochondrial dysfunction. CH25H overexpression had a similar effect on mitochondrial function following LPS treatment (Figure 6). In addition, silencing of CH25H weakened the effect of LPS on mitochondrial function in AC16 cells (Figure 6).

### 3.7 Effect of CH25H on Apoptosis in AC16 Cells

**FIGURE 7:**
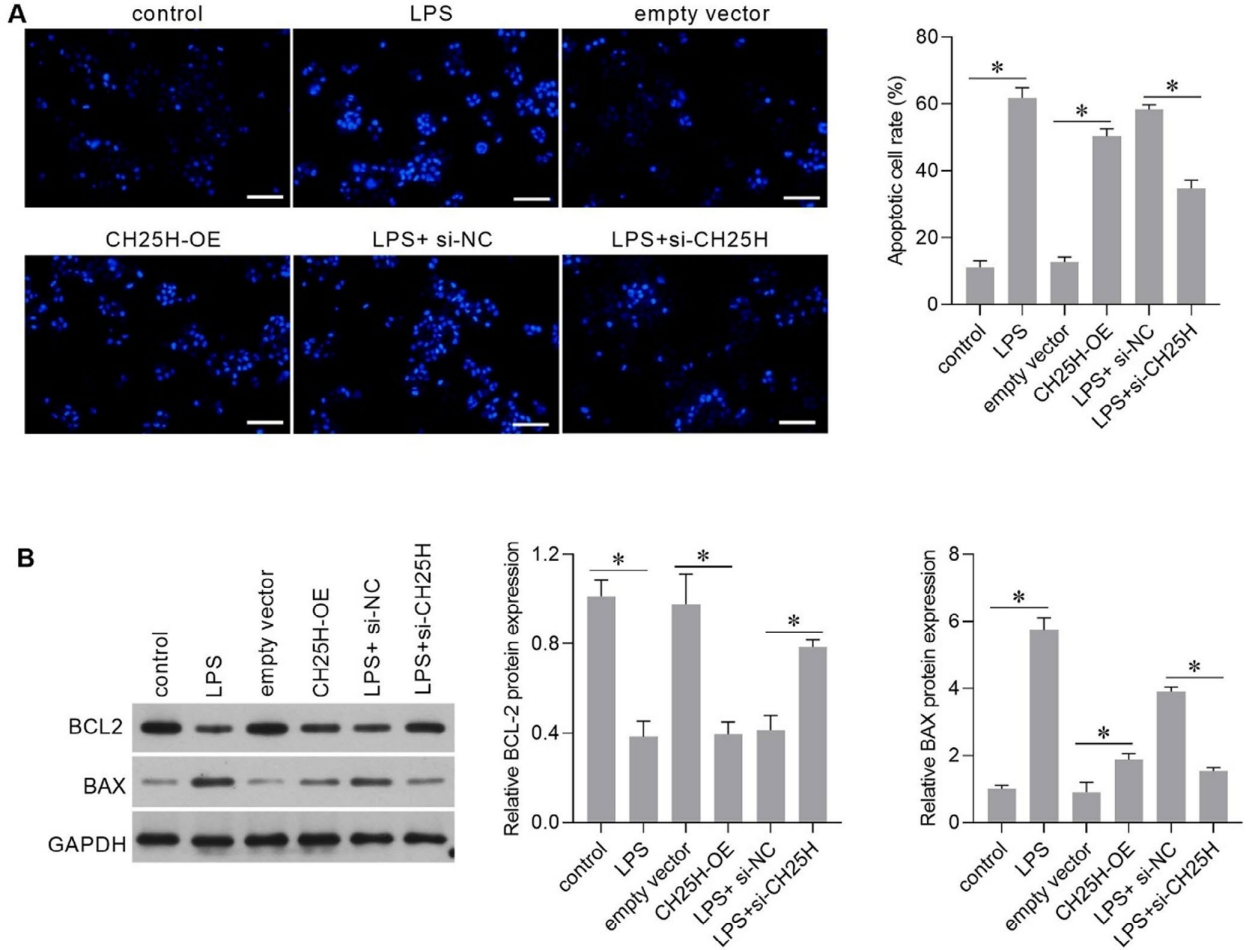
Cholesterol 25‐hydroxylase (CH25H) overexpression enhanced the apoptosis rate and CH25H knockdown attenuated the effects of lipopolysaccharide (LPS) on apoptosis. (A) Hoechst 33258 staining results. The left panel is the representative picture (scale bar = 100 μm), and the right panel is the bar graph of the statistical results of the apoptotic cell rate of each group. (B) B‐cell lymphoma (BCL2) and BCL2‐associated X (BAX) protein levels. The right bar graphs show the expression levels of BCL2 and BAX relative to GAPDH. *p < 0.05, *n* = 3.

Hoechst 33258 staining showed that CH25H overexpression increased the rate of apoptosis (Figure 7A), downregulated the expression of BCL2 (Figure 7B), and upregulated the expression of BAX (Figure 7B), similar to the effects of LPS treatment. Compared with LPS + si‐NC, the apoptotic cell rate decreased (Figure 7A), BCL2 expression was upregulated (Figure 7B), and BAX expression was downregulated in LPS + si‐CH25H cells (Figure 7B). These results showed that CH25H overexpression can induce apoptosis and that CH25H silencing weakened the effect of LPS on apoptosis in AC16 cells. However, CH25H knockdown did not affect apoptosis (Figure S2E) in AC16 cells without LPS stimulation.

### 3.8 Effect of CH25H on NLRP3 Inflammasome Activation in AC16 Cells

**FIGURE 8:**
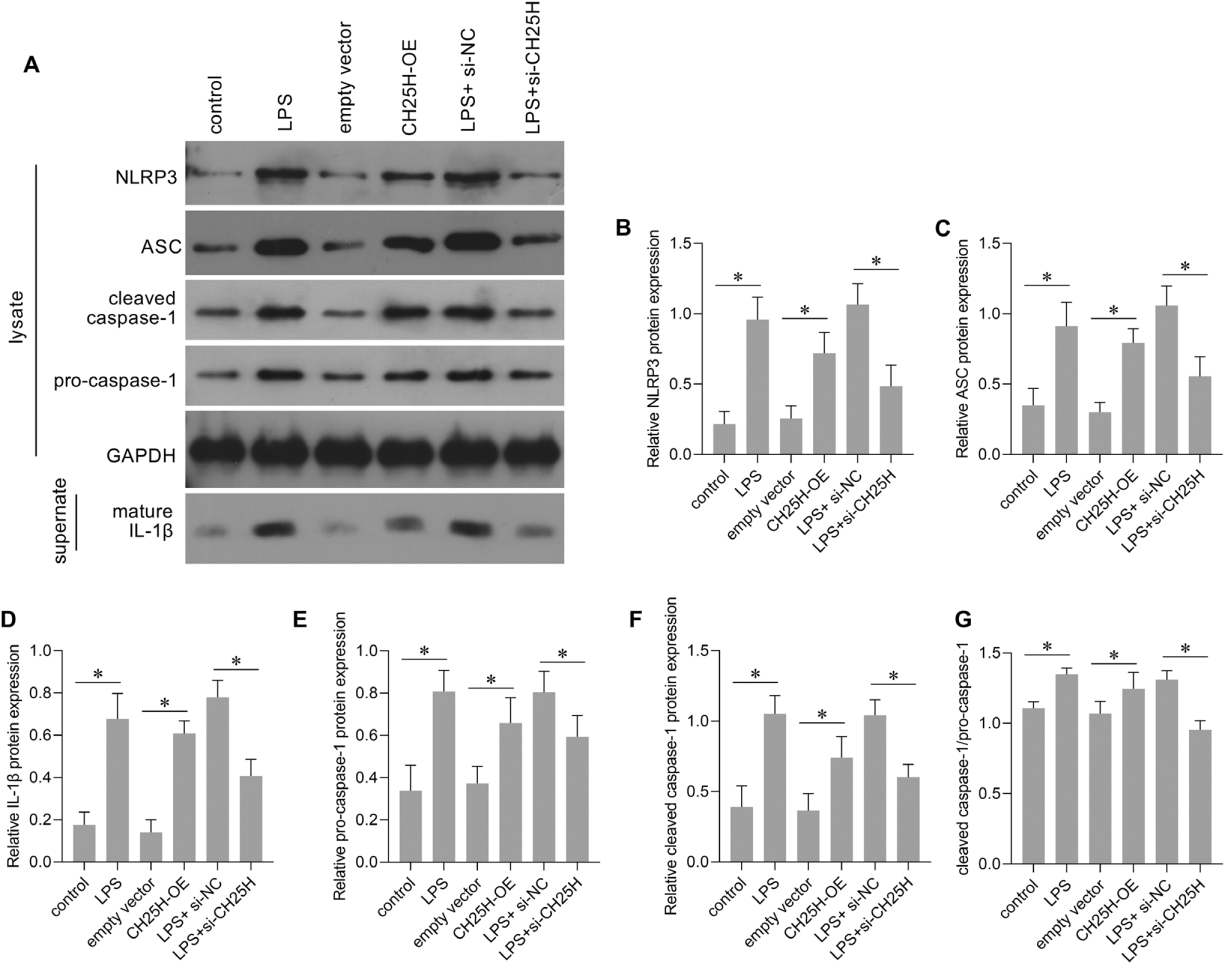
Cholesterol 25‐hydroxylase (CH25H) overexpression upregulated the levels of NOD‐like receptor family pyrin domain‐containing protein 3 (NLRP3), apoptosis‐associated speck‐like protein containing a CARD (ASC), cleaved caspase‐1, pro‐caspase‐1, and interleukin‐1 beta (IL‐1β), and CH25H knockdown attenuated the effects of lipopolysaccharide (LPS) on these proteins. The bar graph in panels B–F shows the statistical result of relative protein expression levels relative to GAPDH. Panel G is the statistical result of cleaved caspase‐1/pro‐caspase‐1 ratio. *p < 0.05, *n* = 3.

NLRP3, ASC, cleaved caspase‐1 (p20/p10), and mature IL‐1β constitute the essential signalling axis of the NLRP3 inflammasome, mediating its assembly, activation, and pro‐inflammatory functions [19]. Compared with the empty vector group, levels of NLRP3, ASC, and IL‐1β were upregulated and the cleaved caspase‐1/pro‐caspase‐1 ratio was increased in the CH25H‐OE group (Figure 8), indicating that CH25H overexpression enhances NLRP3 inflammasome activation. LPS can activate the NLRP3 inflammasome [19], which is consistent with the results of the present study (Figure 8). Compared with the LPS + si‐NC group, levels of NLRP3, ASC, and IL‐1β and the cleaved caspase‐1/pro‐caspase‐1 ratio were decreased in the LPS + si‐CH25H group (Figure 8), indicating that CH25H knockdown blocked the effect of LPS on NLRP3 inflammasome activation in AC16 cells.

### 3.9 Effect of CH25H on NF‐κB Pathway Activation in AC16 Cells

**FIGURE 9:**
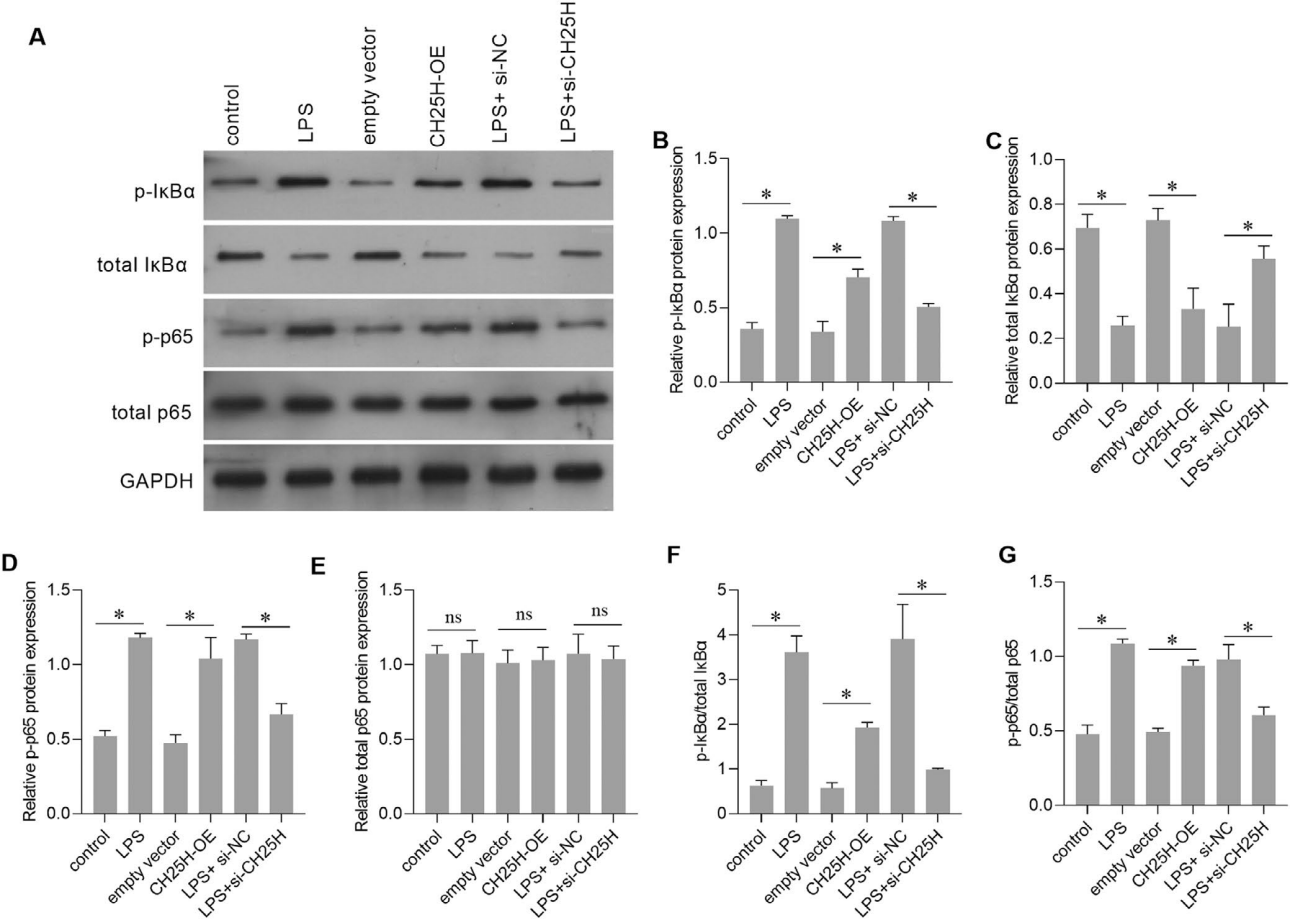
Cholesterol 25‐hydroxylase (CH25H) overexpression activates the NF‐κB pathway and CH25H knockdown attenuated the effects of lipopolysaccharide (LPS) on the NF‐κB pathway. (A) Representative images of Western blot for inhibitor of nuclear factor kappa B alpha (IκBα), phosphorylated IκBα (p‐IκBα), nuclear factor kappa B p65 subunit (p65), and phosphorylated p65 (p‐p65). The bar graph in panels B–E shows the statistical result of relative protein expression levels relative to GAPDH. Panels F and G are the statistical results of p‐IκBα/total IκBα and p‐p65/total p65 ratios. *p < 0.05, and ^ns^ *p* > 0.05, *n* = 3.

IκBα and p65 are two core regulatory proteins in the NF‐κB signalling pathway. In the cytoplasm, IκBα serves as an inhibitor that sequesters p65 [20]. Upon signal stimulation, IκBα undergoes phosphorylation and subsequent degradation, which releases p65, allowing it to translocate into the nucleus and initiate gene transcription. Compared with the empty vector group, p‐IκBα and p‐p65 levels were upregulated and total IκBα level was downregulated in the CH25H‐OE group (Figure 9), indicating that CH25H overexpression activated the NF‐κB pathway. LPS can also activate the NF‐κB pathway (Figure 9). Compared with the LPS + si‐NC group, p‐IκBα and p‐p65 levels were decreased and total IκBα level was increased in the LPS + si‐CH25H group (Figure 9), indicating that CH25H knockdown blocked the effect of LPS on NF‐κB pathway activation in AC16 cells.

### 3.10 NLRP3 Inhibitor Counteracted the Effect of CH25H Overexpression in LPS‐Treated AC16 Cells

**FIGURE 10:**
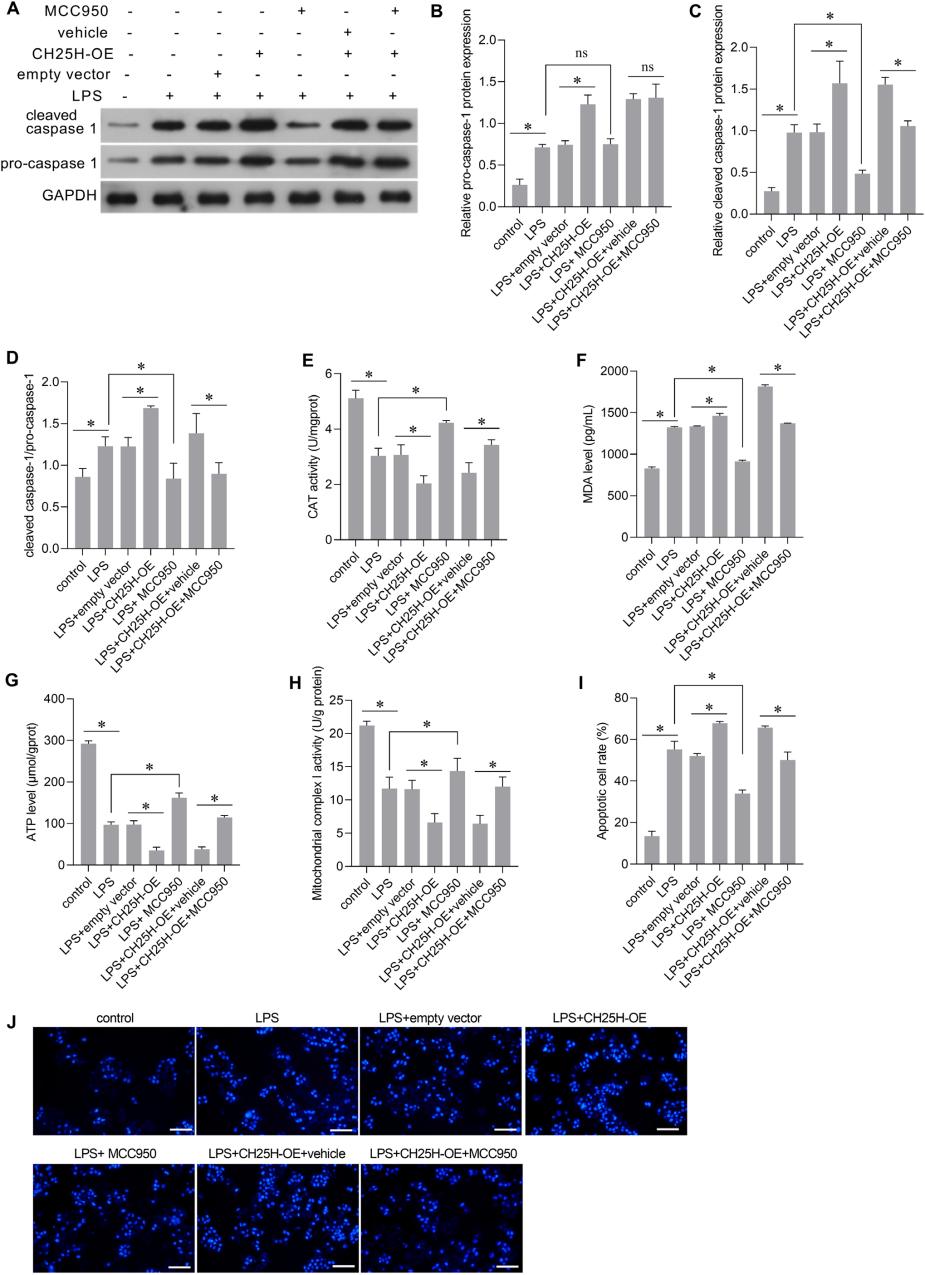
CH25H overexpression exacerbated lipopolysaccharide (LPS)‐induced oxidative stress, mitochondrial dysfunction, and apoptosis, and MCC950 counteracted the effects of CH25H overexpression. (A) Representative images of Western blot for cleaved caspase‐1 and pro‐caspase‐1. (B and C) The statistical result of relative protein expression levels relative to GAPDH. (D) The statistical result of cleaved caspase‐1/pro‐caspase‐1 ratio. (E) Catalase (CAT) activity. (F) Malondialdehyde (MDA) level. (G) ATP level. (H) Mitochondrial complex I activity. (I) The bar graph of the statistical results of the apoptotic cell rate. (J) The representative picture (scale bar = 100 μm) of Hoechst 33258 staining. *p < 0.05, and ^ns^ *p* > 0.05, *n* = 3.

In AC16 cells treated with LPS, CH25H overexpression elevated the level of cleaved caspase‐1 (Figure 10A–D). Further analysis revealed that CH25H overexpression exacerbated oxidative stress (Figure 10E,F), mitochondrial dysfunction (Figure 10G,H), and apoptosis (Figure 10I,J) in LPS‐stimulated AC16 cells. The NLRP3 inhibitor MCC950 effectively suppressed caspase‐1 activation (Figure 10A–D). Compared with the LPS + CH25H‐OE + vehicle group, MCC950 treatment in the LPS + CH25H‐OE + MCC950 group led to reduced CAT activity (Figure 10E), decreased MDA levels (Figure 10F), elevated ATP content (Figure 10G), enhanced mitochondrial complex I activity (Figure 10H), and a lower apoptotic rate (Figure 10I,J). These results indicate that MCC950 treatment counteracted the effects of CH25H overexpression on oxidative stress, mitochondrial dysfunction, and apoptosis in LPS‐treated AC16 cells.

## 4 Discussion

The pathogenesis of SIMD is multifactorial and remains incompletely understood. While inflammatory dysregulation, oxidative stress, and mitochondrial damage have been implicated, the upstream drivers that integrate these pathological processes are not fully defined. CH25H has recently emerged as a potential modulator in inflammatory diseases [5, 6]. However, its expression pattern and functional role in SIMD were unexplored. Therefore, our study was designed to investigate CH25H expression in the myocardial tissue of SIMD mice and the effect and mechanism of CH25H in LPS‐induced cardiomyocyte injury at the cellular level.

Bioinformatics analysis initially revealed a significant upregulation of CH25H in the myocardial tissues of septic animals, which was further validated in both animal models and cellular experiments. To investigate the potential role of CH25H in SIMD, we established an LPS‐induced cardiomyocyte model for functional studies. Although this research was primarily conducted at the cellular level, our findings hold substantial scientific significance. First, we provide the first evidence linking CH25H to SIMD, offering novel mechanistic insights into septic myocardial injury. Second, by confirming the regulatory role of CH25H in LPS‐induced cardiomyocyte damage, this study lays a critical foundation for subsequent in vivo functional investigations and potential clinical translation.

The present study elucidated the roles of CH25H in key pathological processes of LPS‐induced cardiomyocyte damage, including mitochondrial dysfunction, oxidative stress, apoptosis, and NLRP3 inflammasome and NF‐κB pathway activation. These interconnected pathways collectively contributed to the pathogenesis of SIMD [4, 21, 22, 23]. The key manifestations of mitochondrial dysfunction include decreased Δψ, inhibition of oxidative phosphorylation, and decreased ATP synthesis [24, 25]. Mitochondria are also involved in the regulation of cellular redox signalling pathways [26]. ROS are inevitable byproducts of the electron transport chain during mitochondrial respiration [27]. At physiological concentrations, ROS play an important role in cell signalling and maintenance of tissue homeostasis [28]. Under pathological conditions, excessive ROS production can lead to oxidative stress [28]. In sepsis, mitochondrial ROS generation is significantly increased, whereas the production of antioxidant proteins such as CAT, superoxide dismutase, and glutathione peroxidase is greatly decreased, and lipid peroxidation products such as MDA are increased [29, 30, 31]. We found that CH25H overexpression led to significant mitochondrial dysfunction and oxidative stress in AC16 cells. In addition, CH25H overexpression induced apoptosis and NLRP3 inflammasome and NF‐κB pathway activation in AC16 cells. NLRP3 inhibitor counteracted the effect of CH25H overexpression in LPS‐treated AC16 cells. These results indicate that elevated CH25H expression can cause cardiomyocyte damage through NLRP3 inflammasome activation. Moreover, silencing of CH25H weakened LPS‐induced mitochondrial dysfunction, oxidative stress, apoptosis, and NLRP3 inflammasome and NF‐κB pathway activation in AC16 cells. Our results suggest that inhibiting CH25H expression could serve as a potential strategy and target for alleviating LPS‐induced cardiomyocyte damage.

CH25H overexpression increased 25‐HC levels in AC16 cells. 25‐HC induces oxidative stress, mitochondrial dysfunction, apoptosis, and NLRP3 inflammasome activation [12, 17, 18, 32]. Therefore, we hypothesise that CH25H may contribute to LPS‐induced cardiomyocyte damage by regulating 25‐HC levels. To date, no study has reported the role of 25‐HC in LPS‐induced cardiomyocyte damage, especially in SIMD. Therefore, this hypothesis still requires further in vitro and in vivo experiments for validation. Future work will focus on the roles of CH25H and 25‐HC in SIMD through functional validation.

In conclusion, CH25H exhibits high expression in the myocardial tissue of SIMD mice. CH25H may induce cardiomyocyte damage through activating the NLRP3 inflammasome and NF‐κB pathway, while silencing CH25H reduces LPS‐induced cardiomyocyte damage. However, this study has several limitations, including the lack of validation of CH25H function and underlying molecular mechanisms in animal models. Future studies should involve a broader range of cells and animal models to confirm these findings using clinical samples.

## Author Contributions

**Yi‐jiao Men:** conceptualization (lead), funding acquisition (equal), methodology (lead), supervision (equal), writing – original draft (lead). **Hong‐bo Cheng:** methodology (supporting). **Yan‐ling Dong:** methodology (supporting). **Yu Gong:** methodology (supporting). **Ya‐qing An:** methodology (supporting). **Ying‐li Jin:** formal analysis (equal). **Shu‐na Hao:** formal analysis (equal). **Yu Ma:** formal analysis (equal). **Ying‐ping Tian:** conceptualization (equal), supervision (equal), writing – review and editing (equal).

## Funding

This research was supported by grants from the Peking Union Medical Foundation‐Ruiyi Emergency Medical Research Fund (No. 22222012010) and the Medical Science Research Project of Hebei (No. 20211690).

## Ethics Statement

The animal study protocol was approved by the Research Ethics Committee of the Second Hospital of Hebei Medical University (Approval Letter No. 2024‐AE405).

## Consent

The authors have nothing to report.

## Conflicts of Interest

The authors declare no conflicts of interest.

## Supporting information


**Figure S1:** Functional enrichment analysis of 18 DEGs identified using the data of three datasets (GSE153086, GSE40180, and GSE53007). (A) Gene Ontology (GO) enrichment analysis for Biological Process (BP). (B) GO enrichment analysis for Cellular Component (CC). (C) GO enrichment analysis for Molecular Function (MF). (D) Kyoto Encyclopedia of Genes and Genomes (KEGG) pathway enrichment analysis.
**Figure S2:** CH25H knockdown did not affect oxidative stress, mitochondrial dysfunction, and apoptosis in AC16 cells without lipopolysaccharide (LPS) treatment. (A) Catalase (CAT) activity. (B) Malondialdehyde (MDA) level. (C) ATP level. (D) Mitochondrial complex I activity. (D) The bar graph of the statistical results of the apoptotic cell rate. (E) Hoechst 33258 staining results. Left panel is the representative picture (scale bar = 100 μm), and the right panel is the bar graph of the statistical results of the apoptotic cell rate. ^ns^ *p* > 0.05, *n* = 3.

## Data Availability

Data sharing is not applicable to this article, as no datasets were generated or analysed in the current study.
