# Supplementary material for: Silencing of Cholesterol 25‐Hydroxylase Attenuates Lipopolysaccharide‐Induced Cardiomyocyte Damage In Vitro
Source: J Cell Mol Med. 2025 Nov 26;29(22):e70959. doi: 10.1111/jcmm.70959 (PMC12648292; doi:10.1111/jcmm.70959)
Supplement: Supplementary file 1 — Figure S1: Functional enrichment analysis of 18 DEGs identified using the data of three datasets (GSE153086, GSE40180, and GSE53007). (A) Gene Ontology (GO) enrichment analysis for Biological Process (BP). (B) GO enrichment analysis for Cellular Component (CC). (C) GO enrichment analysis for Molecular Function (MF). (D) Kyoto Encyclopedia of Genes and Genomes (KEGG) pathway enrichment analysis. Figure S2: CH25H knockdown did not affect oxidative stress, mitochondrial dysfunction, and apoptosis in AC16 cells without lipopolysaccharide (LPS) treatment. (A) Catalase (CAT) activity. (B) Malondialdehyde (MDA) level. (C) ATP level. (D) Mitochondrial complex I activity. (D) The bar graph of the statistical results of the apoptotic cell rate. (E) Hoechst 33258 staining results. Left panel is the representative picture (scale bar = 100 μm), and the right panel is the bar graph of the statistical results of the apoptotic cell rate. ns p > 0.05, n = 3. [file JCMM-29-e70959-s001.docx]

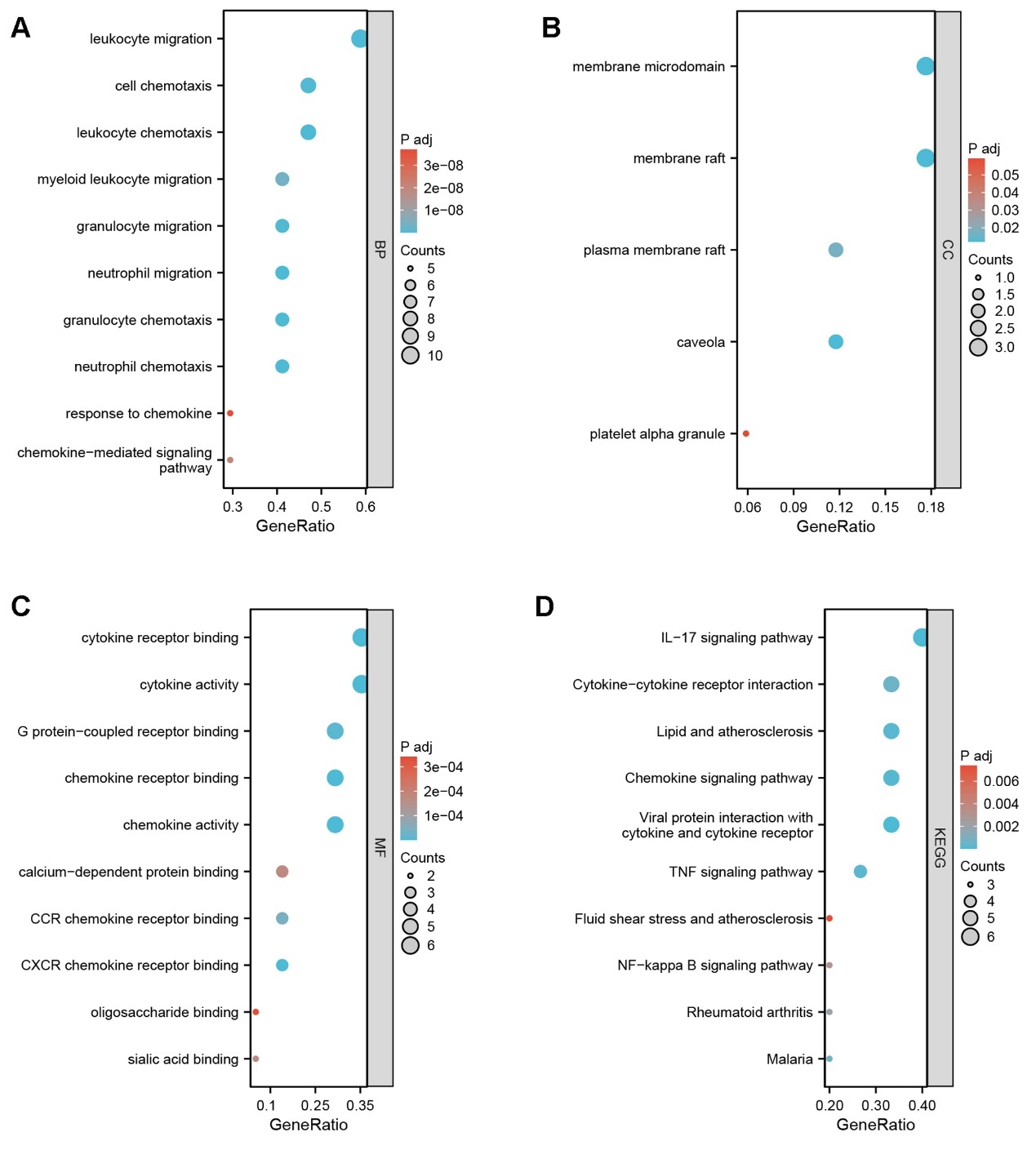


Figure S1. Functional enrichment analysis of 18 DEGs identified using the data of three datasets (GSE153086, GSE40180, and GSE53007). A: Gene Ontology (GO) enrichment analysis for Biological Process (BP). B: GO enrichment analysis for Cellular Component (CC). C: GO enrichment analysis for Molecular Function (MF). D: Kyoto Encyclopedia of Genes and Genomes (KEGG) pathway enrichment analysis.


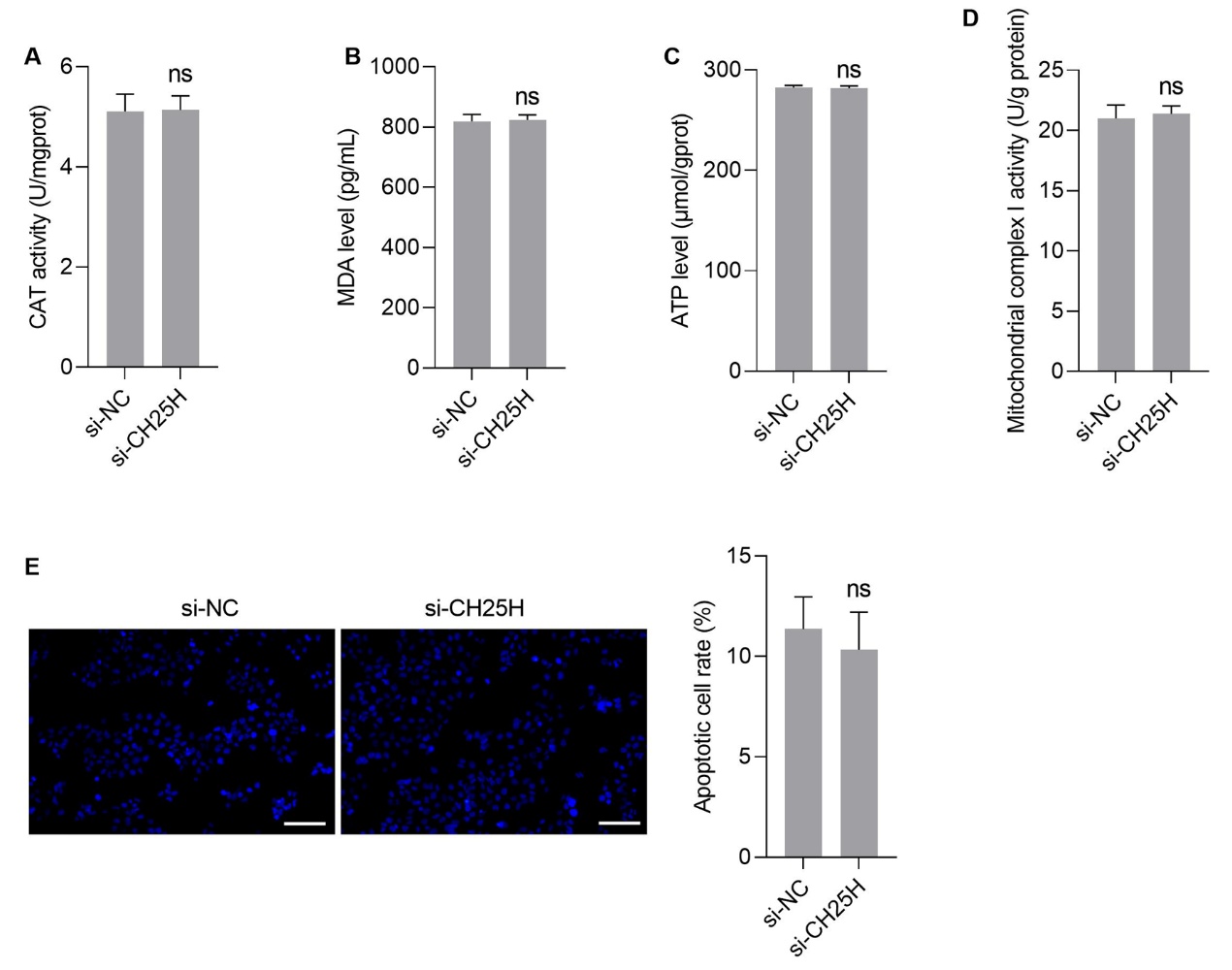


Figure S2. CH25H knockdown did not affect oxidative stress, mitochondrial dysfunction, and apoptosis in AC16 cells without lipopolysaccharide (LPS) treatment. A: Catalase (CAT) activity. B: Malondialdehyde (MDA) level. C: ATP level. D: Mitochondrial complex I activity. D: The bar graph of the statistical results of the apoptotic cell rate. E: Hoechst 33258 staining results. Left panel is the representative picture (scale bar = 100 μm), and the right panel is the bar graph of the statistical results of the apoptotic cell rate. ns means P > 0.05, n=3.
